# Factors associated with *Staphylococcus aureus* nasal carriage and molecular characteristics among the general population at a Medical College Campus in Guangzhou, South China

**DOI:** 10.1186/s12941-017-0206-0

**Published:** 2017-04-11

**Authors:** B. J. Chen, X. Y. Xie, L. J. Ni, X. L. Dai, Y. Lu, X. Q. Wu, H. Y. Li, Y. D. Yao, S. Y. Huang

**Affiliations:** 1 Department of Laboratory, Guangdong Provincial Key Laboratory of Malignant Tumor Epigenetics and Gene Regulation, Sun Yat-Sen Memorial Hospital, Sun Yat-Sen University, Guangzhou, 510120 China; 2 Cross Infection Control Office, Sun Yat-Sen Memorial Hospital, Sun Yat-Sen University, Guangzhou, 510120 Guangdong China; 3 Breast Tumor Center, Sun Yat-Sen Memorial Hospital, Sun Yat-Sen University, Guangzhou, 510120 Guangdong China

**Keywords:** *Staphylococcus aureus*, Nasal carriage, Antimicrobial susceptibility, Medical population, Molecular characteristics

## Abstract

**Background:**

The nasal cavity is the main colonization site of *Staphylococcus aureus* (*S. aureus*) in human body. Nasal carriage may be a strong risk factor for some serious infection. There was still limited information about the nasal carriage for *S. aureus* in south China.

**Methods:**

Sought to determine the prevalence and molecular characteristics of *S. aureus* nasal carriage, 295 volunteers residing on a medicine campus were investigated and sampled the nasal cavity swab. Selected *S. aureus* isolates were carried through molecular analysis, including pulsed-field gel electrophoresis (PFGE), multilocus sequence analysis, staphylococcal cassette chromosome *mec* (SCC*mec*) and virulence gene detection.

**Results:**

A total of 73 *S. aureus* isolates were recovered from separate subjects (24.7%, 73/295), with one methicillin-resistant *S. aureus* (MRSA) isolate (0.3%, 1/295). Among the 73 isolates, 71 isolates were successfully grouped into 13 pulsotypes by PFGE analysis, with profiles A and L the most prevalent; 12 sequence types (STs) were found among the 23 isolates which had similar drug resistant spectrum. ST59, ST188 and ST1 were the most prevalent, accounting for 17.4, 13.0 and 13.0% of all isolates, respectively. The MRSA isolate presented ST8-SCC*mec* III. 56.5% of isolates carried both the staphylococcal enterotoxin A (*sea*) and enterotoxin B (*seb*) genes. 83.6% of the *S. aureus* isolates were resistant to penicillin, all isolates were susceptible to quinupristin/dalfopristin, levofloxacin, teicoplanin and vancomycin. The most common risk factors for *S. aureus* carriage were being male, age ≤30 years, and nasal cavity cleaning habits.

**Conclusions:**

Colonization by *S. aureus* was greater among male and young age (20–30 years) students and those with irregularity nasal cleaning. The *S. aureus* isolates selected were revealed into various sequence types and pulsotypes, indicating molecular heterogeneity among *S. aureus* isolates from the populations in the medical college in Guangzhou.

## Background

*Staphylococcus aureus* (*S. aureus*) can cause nosocomial and community-acquired infections in humans. As a medically pathogen, colonization is a strong risk factor and serious threat to human health. Multiple sites of the human body can be the ecological niche of *S. aureus*, but the main colonization site is the anterior nares [1]. The rates of infection in persistent carriers are higher than others [2]. Besides young age and being male, the main factors to colonize strains were the usage of antibiotics, chronic disease and hospitalization [3–5]. *S. aureus* could spread by contacting with a colonized individual [5]. That may be the reason why individuals without any healthcare-associated risk factors [6, 7] could have led to an increased awareness of community-associated methicillin-resistant *S. aureus* (CA-MRSA). Besides, the *S. aureus* that leads to an invasive infection were in distinguishable from carriage isolates previously isolated from the anterior nares by pulsed-field gel electrophoresis [8, 9] in a patient who gave pulmonary infection. The key to understand the transmission potential of *S. aureus* is to unravel the risk factors for carriage of *S. aureus*. Molecular typing of *S. aureus* is helpful for supporting infection control measures, investigating suspected outbreaks, and preventing nosocomial transmission [10, 11]. Nasal carriage rates were different among races. Moreover, the frequency with which *S. aureus* can be detected in the nose if human individuals, was shown to differ among people of different histocompatibility antigen types (HLA) [12, 13]. It still remains unclear whether carriage rates and risk factors among the Chinese populations that typically lived under crowded conditions are in the same range. And date on *S. aureus* nasal carriage among populations that typically gathered under crowded conditions in Guangzhou is very limited. The medical students need to carry through many experiments which relate to microorganism and go on a field trip to a hospital during their undergraduate career, so they are more likely to expose to the *S. aureus*. Therefore, we sought to determine the prevalence and risk factors of *S. aureus* nasal carriage from students, teachers, retirees among community residents at the campus of Zhongshan School of Medicine, Sun Yat-sen University (SYSU), Guangzhou, South China.

## Methods

### Population and study design

A cross-sectional study was conducted between October 2014 to May 2015, at the campus of Zhongshan School of Medicine, Sun Yat-sen University (SYSU), Guangzhou, South China. All the volunteers from 10 to 76 years old included middle school students, undergraduates, teachers, salesclerks and retirees. The pertinent demographic, medical information and potential factors that are related to *S. aureus* nasal carriage and transmission were collected through a standardized questionnaire. Eleven unqualified surveys whose responses were incomplete were eliminating, 295 nasal swabs were sampled. All volunteers, parents, or guardians signed informed consent documents approving the use of their samples for research purposes, and the study was approved by the Ethics Committee of Sun Yat-Sen Memorial Hospital. [Ethical Approval Number:【2017】伦备第 (01) 号].

### Bacterial strains

Both anterior nares were swabbed by rotating a sterile dry cotton swab 5 times inside the nostril. The samples were immediately stored in Copan eSwab Liquid Amies preservation medium (eSwab Collection and Preservation System, Copan Italia, Brescia, Italy). All swabs were kept at 4 °C, transported at room temperature to the department of bacteriology and processed in 4 h. The swabs were streaked on blood agar plates at 35 °C for 24 h. Gram-positive, β-hemolytic and coagulase positive isolates were confirmed as *S. aureus* using a Vitek^®^ 2 microbial identification system (bioMérieux, Marcy l’ Etoile, France) according to the manufacturer’s instructions. All *S. aureus* were then cultured on MRSA select chromogenic agar. Presumptive MRSA strains, which grew as green colonies on the chromogenic medium, were confirmed by their resistance to cefoxitin and polymerase chain reaction (PCR) for the *mec*A gene [14].

### Antibiotic susceptibility testing

All *S. aureus* isolates were tested for their susceptibility to the following antibiotics: penicillin, erythromycin, clindamycin, cefuroxime, ceftriaxone, cefotaxime, cefoxitin, gentamicin, rifampicin, imipenem, quinupristin/dalfopristin, tetracycline, teicoplanin, vancomycin, trimethoprim/sulfamethoxazole, ciprofloxacin, and levofloxacin. The sensitivity patterns of methicillin-susceptible *S. aureus* (MSSA) and MRSA strains were determined by disk diffusion method according to 25rd informational supplement (M100-S25) which recommended by the Clinical and Laboratory Standards Institute (CLSI; http://clsi.org). The inducible clindamycin resistance was determined by D-test. All disks were obtained from Oxoid Ltd (Oxoid, Basingstoke, England). American type culture collection (ATCC) 25923 *S. aureus* was used as the quality control strain.

### Staphylococcal toxin genes detection

**Fig. 1:**
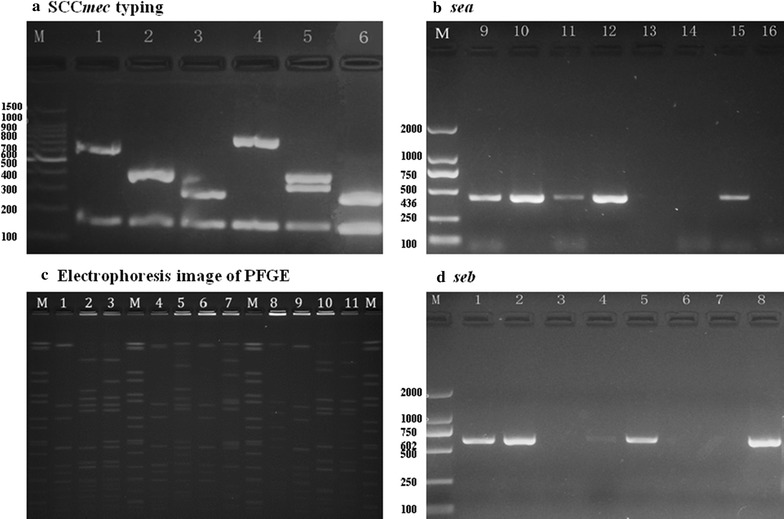
Detection of SCC*mec* types and virulence factor genes. **a** Multiplex PCR assay identifies SCC*mec* types and subtypes I, II, III, IVa, and V. Simultaneously the *mecA* gene was detected. The following DNA templates were used for PCR (by lane): M, marker; 1–5, Control strains (SCC*mec* type I–V); 6, *S. aureus* strains isolated from the volunteers in the study. **b** PCR assay identifies *sea* gene. The following DNA templates were used for PCR (by lane): M, marker; 9, positive control; 10–16, *S. aureus* strains isolated from the volunteers in the study. **c** Electrophoresis image of Pulsed field gel electrophoresis. The following DNA templates were used for electrophoresis (by lane): M, The positive control strain *Salmonella ser. Braenderup standard strain H9812*; 1–11, *S. aureus* strains isolated from the volunteers in the study. **d** PCR assay identifies *seb* gene. The following DNA templates were used for PCR (by lane): M, marker; 1, positive control; 2–8, *S. aureus* strains isolated from the volunteers in the study

The Panton-Valentine leukocidin (*pvl*) and the staphylococcal enterotoxin A (*sea*) and enterotoxin B (*seb*) genes were detected by PCR as previously described [14, 15] (Fig. 1).

### Staphyloccoccal cassette chromosome *mec* (SCC*mec*) typing

Multiplex PCR was performed for SCC*mec* typing of MRSA isolates by using eight unique pairs of primers for SCC*mec* types and subtypes I, II, III, IVa, IVb, IVc, IVd, and V, as described previously [16] (Fig. 1). Positive control strains for SCC*mec* types I (NCTC 10442), II (N315), III (85/2082), and IVa (JCSC 4744), were kindly provided by Dr. Fangyou Yu of the Department of Laboratory Medicine, the First Affiliated Hospital of Wenzhou Medical College.

### Multilocus sequence typing (MLST)

MLST was performed using previously described primers and conditions [17], the sequence types (STs) was obtained through the website http://saureus.mlst.net and the clonal complexes (CCs) were defined by analyzing cluster related STs with the eBURST software (http://eburst.mlst.net/v3/enter_data/single). A neighbor-joining tree was constructed from the sequence data using MEGA version 6.06 [11]. STs that grouped together with ≥70% bootstrap support were considered part of the same CC.

### Pulsed-field gel electrophoresis (PFGE)

PFGE was performed using *SmaI* as described previously [18] (Fig. 3). The relatedness of the strains were determined according to the criteria of Tenover et al. [19]. The isolates with >75% similarity were clustered in patterns. The dendrograms were generated by analyzing the electrophoretogram with BioNumerics version 5.01 statistical software, according to a simple matching coefficient and the unweighted pair group method with the arithmetic mean (UPGMA) algorithm. The same PFGE patterns were grouped as a pulsotype and assigned alphabetically (A, B, C etc.).

### Statistical analysis

Frequencies were obtained and proportions were calculated for categorical variables. The only continuous variable, age, was transformed into a categorical variable using the quartiles of the frequency distribution (≤20, >20–30, >30–50, >50 years). Categorical variables were compared using the Chi square test or the Fisher exact test. Odds ratios (OR), 95% confidence intervals (CI), and *P* values were calculated. A *P* value of ≤0.05 was considered statistically significant. Univariable logistic regression models were applied to determine independent risk factors. Multiple logistic regression analysis was carried out by stepwise backward selection of variables with biological plausibility and a significance level <0.10 for entry into the model. Statistical comparisons were performed with SPSS (PASW Statistics 18) software (IBM, Armonk, NY). All susceptibility data were analyzed using WHONET software, version 5.6.

## Results

### Nasal colonization with *S. aureus*

A total of 295 volunteers were enrolled onto this study. The median age of the participating volunteers was 30.0 years (range 10–76 years), and 45.8% (135/295) were male. Distributions of *S. aureus* carriers and non-carriers stratified by population characteristics and variables associated with *S. aureus* carriage in the univariate analysis were shown in Table 1. The overall prevalence of *S. aureus* carriage was 24.7% (73/295). The nasal carriage of *S. aureus* was 32.6% (44/135) in males, which is higher than 18.1% (29/160) in females (OR 2.04, 95% CI 1.27–3.79). The difference between nasal carriage in male and female was statistically significant (*P* < 0.05). Highest nasal carriage, 33.1% (41/124) (OR 3.30; 95% CI 1.12–9.75) of *S. aureus* was recorded in the age group of ≤20 years, followed by 26.5% (18/68) (OR 2.71, 95% CI 1.15–7.51), 17.0% (8/47) (OR 1.91, 95% CI 0.44–4.91), and 10.7% (6/56) in the age groups of 20–30 years, 30–50 years and >50 years. There was statistical significant difference (*P* < 0.05) between ages <20 years and 20–30 years. The corresponding rates were 13.0% (13/100) and 30.4% (60/194) (OR 0.34, 95% CI 0.17–0.65) between those who clean their nasal frequently or occasionally, respectively, and there was statistical significant difference (*P* < 0.05). In multiple logistic regression analysis, nasal carriage of *S. aureus* was also significantly associated with male, age ≤20 years, and regular cleaning of the nasal cavity.

**Table 1:** Univariate and multivariate analysis of risk factors associated with *S. aureus* nasal carriage among 295 volunteers at the campus of Zhongshan School of Medicine, Sun Yat-sen University, Guangzhou

Characteristic	The healthy people (n = 295), n (%)					
	Carriers (n = 73) n (%)	Non-carriers (n = 222) n (%)	Univariate		Multivariate logistic	
			*P* value	OR (95% CI)	*P* value	OR (95% CI)
Sex						
Male	44 (32.6)	91 (67.4)	0.005	2.04 (1.27–3.79)	0.021	2.51 (1.29–3.91)
Female	29 (18.1)	131 (81.9)				
Age, years						
≤20	41 (33.1)	8 (66.9)	<0.001	0.25 (0.11–0.54)	0.01	3.30 (1.12–9.75)
>20–30	18 (26.5)	50 (73.5)	0.021	0.31 (0.11–0.84)	0.041	2.71 (1.15–7.51)
≥30–50	8 (17.0)	39 (83.0)	0.312	0.56 (0.18–1.74)	0.451	1.91 (0.44–4.91)
≥50	6 (10.7)	50 (89.3)		1		1
Antibiotic use in past 1 month						
Yes	4 (20.0)	16 (80.0)	0.612	0.75 (0.24–2.31)		
No	69 (25.1)	206 (74.9)				
Regular contact nasal cavity cleaning						
Yes	13 (13.0)	87 (87.0)	0.001	0.34 (0.17–0.65)	<0.001	0.29 (0.15–0.56)
No	59 (30.4)	135 (69.6)				
Hospitalization in past one year						
Yes	4 (36.4)	7 (63.6)	0.369	1.78 (0.51–6.27)		
No	69 (24.3)	215 (75.7)				
Underlying disease						
Yes	11 (36.7)	19 (63.3)	0.891	1.05 (0.50–2.21)		
No	62 (23.4)	203 (76.6)				
Household member working in Medical Institutions						
Yes	14 (26.4)	39 (73.6)	0.689	1.15 (0.58–2.27)		
No	59 (24.4)	183 (75.6)				

Underlying disease: hypertension, diabetes, chronic rhinitis, urticaria, hyperthyroidism

*OR* odds ratio, *CI* confidence interval

### Antibiotic susceptibility

**Fig. 2:**
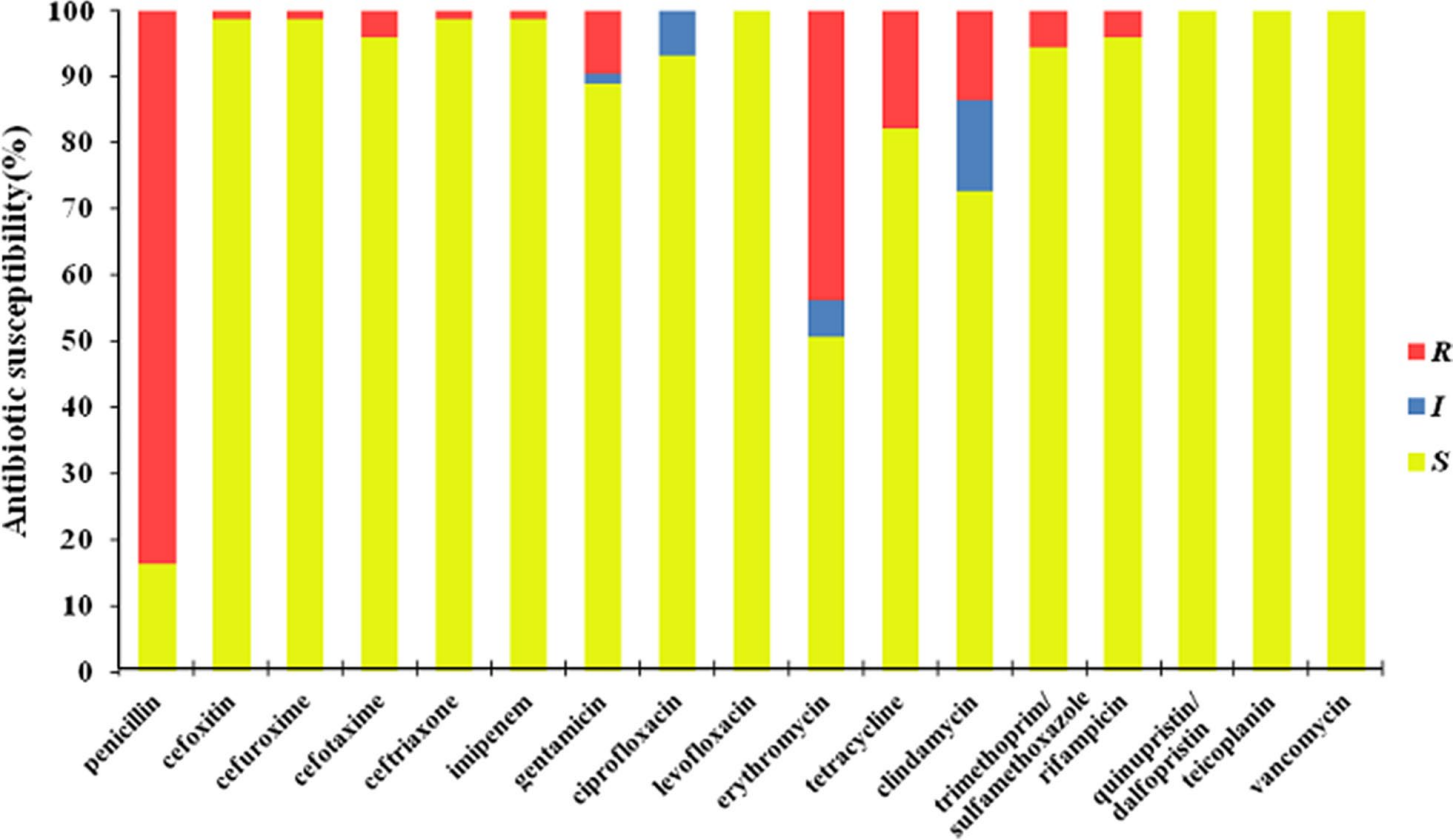
Antibiotic susceptibility profiles of 73 *S. aureus* isolates

Susceptibility pattern of *S. aureus* to various antibiotics is shown in Fig. 2. Among the 73 *S. aureus* isolates, 61 (83.6%) were resistant to penicillin and 32 (43.8%) to erythromycin. The isolate resistant to tetracycline and clindamycin was found in 13 (17.8%) and 10 (13.7%) isolates respectively. Rates of resistance to cefuroxime, ceftriaxone, cefotaxime, cefoxitin, trimethoprim/sulfamethoxazole, gentamicin, rifampicin, and imipenem were <10%. All isolates were susceptible to levofloxacin, quinupristin/dalfopristin, teicoplanin, and vancomycin. Among 73 *S. aureus* isolates, only one isolate (1.4%, 1/73) was resistant to cefoxitin and further confirmed to be MRSA detecting *mec*A gene by PCR screening. It was resistant to penicillin, cefuroxime, ceftriaxone, cefotaxime, erythromycin, clindamycin, tetracycline, and gentamycin. The isolate was separated from a 17-year-old female middle school student, who had be in hospital for some days and taken antithyroid drugs because of thyroid problem in past half a year.

### Detection of toxin genes and SCC*mec* typing

Detection of toxin genes and SCC*mec* typing were shown in Fig. 1. Only one isolate was clearly typed harbored SCC*mec* III. Among all the *S. aureus* isolates, 56.5% detected both *sea* and *seb*, 26.1% detected only *sea* and 13.0% detected only *seb*. And the MRSA isolate was only detected *seb*. Seven isolates that detected both *sea* and *seb* were found separated from the students who came from the same class. There was not statistical significant difference (*P* > 0.05) among age, gender and profession in detection of enterotoxin gene. Interestingly, the *pvl* gene was not detected among all the *S. aureus* isolates in this study.

### MLST and PFGE typing

**Fig. 3:**
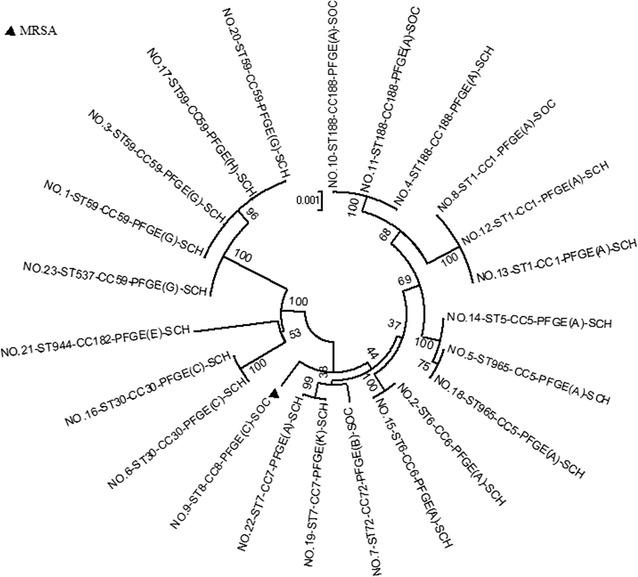
A neighbour-joining tree reveals phylogenetic relationships of 23 *S. aureus* strains isolated from the volunteers in the study. The neighbor joining tree was based on the concatenated sequences of each of the 23 sequence types noted in the combined dataset, as determined using the *S. aureus* MLST database (http://saureus.mlst.net/) and implemented in MEGA v6.06 using Kimura-2-parameter distances. The relationships shown were based on 1000 re-samplings for bootstrapping. Each clonal complex (CC) is composed of STs that cluster with a ≥70% bootstrap confidence value. Bsides, Bayesian phylogram indicating the evolutionary relationships of *S. aureus* strains analyzed in this study. PFGE, pulsed-field gel electrophoresis; SCH, the volunteers who work or study in the college; SOC, the volunteers who work beside the college

Twenty-three *S. aureus* isolates with similar drug-resistant spectrum were revealed 12 different sequence types. Among these, ST59, ST188 and ST1 were the most prevalent, accounting for 17.4% (4/23), 13.0% (3/23) and 13.0% (3/23), respectively. Other ST types were ST965, ST30, ST6, ST7, ST5, ST8, ST72, ST537 and ST944. With the eBURST software, 10 CCs were identified. The main CC was CC59 (n = 5), followed by CC188 (n = 3), CC1 (n = 3), CC5 (n = 3), CC6 (n = 2), CC7 (n = 2), CC30 (n = 2), CC8 (n = 1), CC72 (n = 1) and CC182 (n = 1). The MRSA isolate was revealed ST8 (Table 2). Of the 73 *S. aureus* isolates collected, 71 isolates consisted of 13 different pulsotypes, while two isolates were untypable. Patterns were classified from A-M, each defining a clone in according with the previously reported interpretive criteria [19]. The most prevalent profiles were A (50.7%, 36/71) and L (18.3%, 13/71), the MRSA isolate belonged to pulsotype C. 69.4% (25/36) of the isolates separated from students were came from the same class in profiles A, and 84.6% (11/13) in profile L (Fig. 3).

**Table 2:** Demographic characteristics and molecular features of 23 cases with *S. aureus* carried

Case	Age	Gender	Profession	Resistance profile	*pvl*	*sea/seb*	ST	CC	PFGE
1	20	Female	SCH	PEN, GEN, ERY, TCY, CLI, SXT	−	+/+	59	59	G
2	23	Male	SCH	PEN	−	±	6	6	A
3	22	Male	SCH	PEN, ERY, TCY, CLI	−	±	59	59	G
4	21	Male	SCH	NR.	−	−/+	188	188	A
5	20	Female	SCH	PEN, CTX	−	+/+	965	5	A
6	62	Female	SCH	PEN, ERY	−	+/+	30	30	C
7	23	Female	SOC	PEN	−	+/+	72	72	B
8	70	Female	SOC	PEN, CIP, CLI	−	+/+	1	1	A
9	17	Female	SOC	PEN, FOX, CXM, CTX, CRO, IPM, CIP,GEN, ERY, TCY, CLI	−	−/+	8	8	C
10	65	Female	SOC	PEN	−	−/+	188	188	A
11	62	Female	SOC	PEN	−	−/−	188	188	A
12	53	Male	SCH	PEN, CLI	−	+/+	1	1	A
13	24	Female	SCH	PEN, CLI	−	±	1	1	A
14	13	Female	SCH	GEN, ERY, CLI, SXT	−	+/+	5	5	A
15	14	Male	SCH	ERY	−	±	6	6	A
16	14	Male	SCH	PEN, GEN, ERY	−	±	30	30	C
17	14	Female	SCH	PEN, ERY, TCY, CLI	−	±	59	59	H
18	14	Male	SCH	PEN, GEN, ERY, TCY	−	+/+	965	5	A
19	13	Female	SCH	PEN, GEN, ERY, CLI, SXT	−	+/+	7	7	K
20	14	Male	SCH	NR.	−	+/+	59	59	G
21	13	Female	SCH	ERY	−	+/+	944	182	E
22	14	Male	SCH	NR.	−	+/+	7	7	A
23	14	Male	SCH	NR.	−	+/+	537	59	G

*SCH* the volunteers who work or study in the college, *SOC* the volunteers who work beside the college, *pvl* Panton-Valentine leukocidin gene, *sea* staphylococcal enterotoxin A gene, *seb* staphylococcal enterotoxin B gene, *ST* sequence type, *CC* clonal complexe, *PFGE* pulsed-field gelelectrophoresis, *PEN* penicillin, *FOX* cefoxitin, *CXM* cefuroxime, *CTX* cefotaxime, *CRO* ceftriaxone, *IPM* imipenem, *TCY* tetracycline, *CIP* ciprofloxacin, *CLI* clindamycin, *ERY* erythromycin, *GEN* gentamicin, *SXT* trimethoprim/sulfamethoxazole, *NR.* the isolate is sensitive to all the antibiotics, ***−*** negative, *+* positive

## Discussion

*Staphylococcus aureus* nasal carriage is not infrequent in China but few reports on the prevalence and the risk factors of *S. aureus* nasal carriage are found. The findings of this study are of significance to understand *S. aureus* nasal colonization dynamics within the special community, and to design strategies to prevent *S. aureus* infection and dissemination. *S. aureus* nasal carriage is a global phenomenon among healthy population. But detection rate of *S. aureus* nasal carriage is different in different area. The overall prevalence of *S. aureus* carriage was 24.7% in this study. Only one *S. aureus* was founded as MRSA (1.4%). Compared with the general population, this coincides with the recorded prevalence among 2448 healthy people from Beijing and Harbin in Northern China (16.5%), of which 0.3% were MRSA and in adults in community settings in Taiwan (22.1%) [20, 21]. Besides, previous studies revealed a similar nasal carriage rate (15.4–23.1%) and low prevalence of MRSA colonization (3.0–9.4%) in Chinese medical students from different regions. Another study also revealed a similar nasal carriage rate (20%) with no MRSA strains identified in military volunteers from Beijing [22]. In addition, it also coincides to multiple reports of CA-MRSA infections on college and high school campuses, with a concentration of cases occurring among student athletes [23]. But, in contrast, the nasal carriage rate of MRSA colonization was found to be 11.6% in a cohort of healthy children aged ≤14 years in community settings in Taiwan over a 5-year period [24], which was higher than that in this study. Compared with other nations, the detection rate of *S. aureus* nasal carriage is lower than that in America and Europe (20–30%) [25]. which coincides to the report that the detection rate of MRSA among adults is 1% in Thailand [26], 0.2% in Northern Europe and 3% in Northern America [27], but is lower than 8.6% in Mexico [28]. In addition, the analogous report that the detection rate of *S. aureus* at Wenzhou Medical College in Wenzhou was 15.4%, of which 3.0% were MRSA [29]. Besides, the study from Brasil reported that in a medical student community, the detection rate of *S. aureus* and MRSA were 20.6 and 3.4% [30]. In contrast, the detection rate of *S. aureus* in this study is higher than the analogous report, but the detection rate of MRSA is lower. In conclusion, though the special community living or working in crowded conditions are more likely to expose to *S. aureus* colonization and infection [29], but the nasal carriage detection rate of MRSA is not higher.

Previous studies have found that young age, male sex, chronic sinusitis, nonuse of antibiotics, the length of hospital stay, less education, and drug use were the risk factors associated with *S. aureus* colonization [31, 32]. Yan et al. [20] found that the populations that typically lived under crowded conditions would have higher opportunities for transmission and non-Han Chinese, youth male and chronic disease were the most possibly risk factors of nasal *S. aureus* carriage in healthy population. Higher *S. aureus* carriage rates were also found in men, individuals with obesity and children in Chinese medical college campus [29]. Another study reported that insufficient immunity, crowds or closed contacts, and inactivity was an ideal setting for *S. aureus* [22]. In Taiwan, a study also found that crowded environments, such as living with a greater number of children and attending day care, significantly increased the risk of MRSA colonization [24]. Our study also found that higher *S. aureus* carriage rates were associated with being male, young age and nonstandard nasal cleaning habits. Previous studies have reported that predisposes healthy individuals and transplant recipients to *S. aureus* nasal carriage with *S. aureus* will be affected by the human leukocyte antigen (HLA) DR3 antigen [13, 33]. Thus, gender specific frequencies of HLA haplotypes may lead to differential susceptibilities between male and female. However, HLA DR3 haplotype frequencies among the carriers have not been investigated. Cleaning the nasal cavity with regularity can protect against nasal colonization by *S. aureus* [34]. According to the standardized questionnaire we found that female volunteers were more likely to clean the nasal cavities with regularity, so it may be an important reason why the nasal carriage of *S. aureus* in males was higher than in females. So it was absolutely essential to clean the nasal cavity with regularity among those who were exposed to higher *S. aureus* carriage rates. Unfortunately, we could not find any difference between nasal carriage in the healthy people and the population under chronic disease was statistically significant because the number of the population under chronic disease was too small. It was observed that *pneumococcal* competition at the Youth of life would lead to a negative correlation for the cocolonization of *S. aureus* and *Streptococcus pneumonia* [35]. Besides, the volunteers whose ages ≤20 years were more likely to study together for more than 7 h per day, so they had more closely intimate contact with each other. What’s more, compared with adults, they would not clean the nasal cavity with regularity. So it may be the explanation for the phenomenon that higher *S. aureus* carriage rates were associated with young age.

As known, the object of study to MLST was always MRSA but not MSSA. CC5, CC8, CC188, ST398 and CC59 [20, 21, 36, 37] were the major CCs among the *S. aureus* strains separated from both nasal and clinical according to those previous studies from China. But ST7 and CC188 occurred quite a lot in the recent Chinese studies, which indicated that the majority of the MSSA clones observed in China are globally distributed [36, 38]. In this study, 23 *S. aureus* isolates were separated into 12 STs, which belonged to 10 CCs, including 4 major CCs: CC59, CC188, CC1 and CC5, respectively. This coincides with the study to MLST of the *S. aureus* strains separated from nasal among healthy adults in China [20]. And ST59, which belonged to CC59, was the major ST of CA-MRSA in China [36], what’s more, the major type of CA-MRSA in Taiwan was ST59-SCC*mec*V (*pvl*-positive), followed by ST59-SCC*mec*IV (*pvl*-negative), but 46–59% MRSA isolates that separated from the nasal of healthy adults belonged to ST59-SCC*mec*IV (*pvl*-negative). Interestingly, the MRSA isolate detected in this study belonged to ST8-SCC*mec*III (*pvl*-negative), which was the endemic genotype of hospital-acquired MRSA (HA-MRSA) but not CA-MRSA in China [39]. This finding lends further support to the notion that HA-MRSA was going to instead of CA-MRSA by nasal carriage transmission because the parasitifer of the MRSA isolates had a history of exposure to a hospital environment.

PFGE electrophoresis could separate large fragments of DNA, so it was recommended as the standard technique for *S. aureus* genotyping, using for pathogen traceability research and the investigation of hospital infection outbreak [40]. In this study, the *S. aureus* isolates separated from the population in the same class had the same PFGE pulsotype, which indicated that the *S. aureus* may spread from carrier to non carrier by nasal.

Panton-Valentine leukocidin [41, 42], encoded by *pvl* gene, was a toxin that lyses leukocytes in a receptor-dependent fashion and associated with skin and soft tissue infection. Previous study showed that the acquisition of a mobile genetic element carrying the genes coding for *pvl* gene was mainly based on the finding that most initially found CA-MRSA clones *pvl*-encoding genes, while HA-MRSA commonly do not [41]. But the role of *pvl* gene in the pathogenesis of CA-MRSA infections, in particular skin infection as the most common manifestation of CA-MRSA disease, is still controversial [43]. Interestingly, the *pvl* gene was not detected among all the *S. aureus* isolates in this study. It coincidesed with the study by Sanchini et al. [44], who collected 18 CA-MRSA strains from all over Italy but only found one of these tested positive for *pvl* gene.

*Staphylococcus aureus* enterotoxins (SE) are major causes of staphylococcal food poisoning [45]. Staphylococcal enterotoxin A and enterotoxin B genes, which were associated with severe disease such as necrotizing soft tissue infections, were confirmed as the most abundant toxin genes in clinical *S. aureus* isolates from patients and children in China [46, 47]. Previous reports have found that isolates from patients with bacteremia always detected *sea* and *seb* was identified in isolates from sputum samples, and the most abundant toxin genes in clinical *S. aureus* isolates from patients and children in China were *sea* and *seb* [46, 47]. Our recent study have shown that 56.5% CA-MRSA and 61.5% HA-MRSA carried the *sea* gene. However, *seb* gene was only detected in CA-MRSA isolates (52.2%) [48]. Another previous investigation has demonstrated that a significantly higher proportion of *sea* gene-positive isolates came from the community residents compared with the healthcare workers (26.1 vs. 5.6%, *P* = 0.024) [34]. The important finding of the current study was the high rate of detection of the enterotoxin genes in *S. aureus* (56.5% detected both *sea* and *seb*), which indicated that *S. aureus* carriers are at risk of autoinfection.

Broader concerns should be paid on the antimicrobial resistance problems in *S. aureus*. *S. aureus* strains can differ in their susceptibility profiles [49, 50]. In this study, we found that there was a high rate of resistance against penicillin and erythromycin. The reason might be the excessive use of penicillin and macrolides [49, 50]. Fortunately, most of the isolates remained sensitive to the majority of antibiotics, gentamicin, rifampicin, trimethoprim/sulfamethoxazole, quinupristin/dalfopristin and teicoplanin. Therefore, formulating more strategies for rational use of antibiotics are urgently required.

There are limitations to note about the current work. Most importantly, as a cross-sectional study, to detect variations in colonization patterns, e.g. persistent carriers, intermittent carriers, or non-carriers was unpractical. Secondly, because the number of volunteers is limit, recruitment of subjects from the college may mean that the results are not generalizable to the population of the medicos in Guangzhou as a whole, despite the good cross sections of age and occupation among the test subjects. Thirdly, sampling only the nostrils without including other body parts may underestimate the frequency of MRSA carriage overall [50].

## Conclusions

This study showed that being male, young age (20–30 years) and irregularity nasal cleaning are more likely to be colonized by *S. aureus*. The finding lends further evidence of molecular heterogeneity among *S. aureus* isolates from the populations in the medical college in Guangzhou.
